# A Case of Fatal Gangrene of the Fauces, Caused or Attended by Worms

**Published:** 1853-05

**Authors:** William Boyd

**Affiliations:** Rock Island, Texas


**Art.** III.—*A Case of Fatal Gangrene of the Fauces, caused or attended by Worms.* By William Boyd, M. D., of Rock Island, Texas.

Burke, a black man, aged forty-five years, first attracted the attention of his master on the 15th of September, 1852, by bleeding at the nose. On entering his room, I perceived an offensive odor, and on injecting a solution of zinc into the nostrils for the bleeding, several worms were discharged, bearing a resemblance to the common maggot. By employing warm water and soap, a considerable number were brought away; the hemorrhage was arrested by the zinc. Having found calomel and sweet oil efficacious in destroying maggots in the wounds of cattle, I directed an injection to be used consisting of these articles. The next day a number of worms were discharged.

The treatment was continued, and on the 5th, the patient was so far relieved, that he walked a mile or two to church.

7th.—I was called to the patient again, and found him complaining of a dull pain in the head, very restless, pulse hard and quick, bleeding at the nose and mouth. Enemata were used to move his bowels, and the injections into the nose, as before.

8th.—Patient worse. Considerable fever, complains of pain in his head and neck. Tartar emetic, in small doses. Injections of spirits turpentine and calomel into the nostrils.

9th.—Nothing except slight swelling detected in the fauces. Symptoms appeared slightly improved.

10th. — Breathing stertorous; pulse small, quick, stupor, perspiration. On examination, soft palate found in a state of sphacelus, and when this was excised, the cavity was discovered to be full of worms. After their removal, his throat having been cleansed, patient felt better, and breathed with more freedom.

On the 11th, he died.

*Post-Mortem.*—The brain and membranes were found engorged with black blood. The branches of the olfactory nerve were dark from the presence of the same. The cribriform plate of the ethmoid bone presented a dark appearance, and other parts of this bone were soft, and filled with a dark grumous matter. The whole posterior fauces were in a state of mortification, and at one point the cervical vertebræ were nearly denuded.

November, 1853.

